# Diethyl indolizine-1,3-dicarboxyl­ate

**DOI:** 10.1107/S1600536810050919

**Published:** 2010-12-15

**Authors:** Wei-Jin Gu, Jin Zhuang, Yu-Liang Jiang, Bing-Xiang Wang

**Affiliations:** a Department of Applied Chemistry, Nanjing Normal University, Nanjing 210097, People’s Republic of China

## Abstract

The title compound, C_14_H_15_NO_4_, was prepared by a 1,3-dipolar cyclo­addition from *N*-(eth­oxy­carbonyl­methy)pyridinium bromide and ethyl acrylate. The –CO_2_ side chains form dihedral angles of 0.2 (3) and 2.4 (3)° with respect to the ring system. In the crystal, two neighbouring mol­ecules form a dimer through weak C—H⋯O interactions. The dimers form a three-dimensional structure *via* further weak C—H⋯O inter­actions.

## Related literature

For synthetic procedures, see: Teklu *et al.* (2005 ▶), Wang *et al.* (2000 ▶). For the pharmaceutical use of related compounds, see: James *et al.* (2008 ▶), Tukulula *et al.* (2010 ▶). For the use of related compounds as organic fluorescence probes, see: Shen *et al.* (2006 ▶, 2008 ▶).
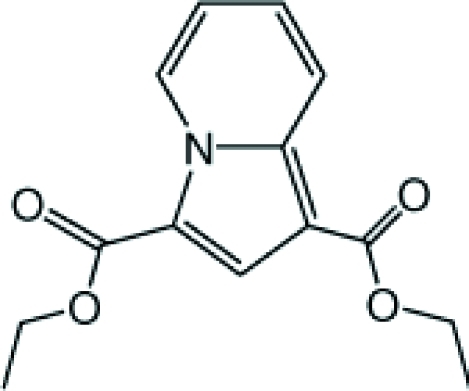


## Experimental

### 

#### Crystal data

C_14_H_15_NO_4_
*M* *_r_* = 261.27
Monoclinic, 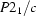

*a* = 7.941 (2) Å
*b* = 19.700 (4) Å
*c* = 8.622 (2) Å
β = 101.770 (3)°
*V* = 1320.5 (5) Å^3^
*Z* = 4
Mo *K*α radiation
μ = 0.10 mm^−1^
*T* = 291 K
0.30 × 0.26 × 0.24 mm

#### Data collection

Bruker SMART APEX CCD diffractometer
Absorption correction: multi-scan (*SADABS*; Bruker, 2000 ▶) *T*_min_ = 0.972, *T*_max_ = 0.977
7930 measured reflections
2400 independent reflections
1567 reflections with *I* > 2σ(*I*)
*R*_int_ = 0.039

#### Refinement

*R*[*F* ^2^ > 2σ(*F* ^2^)] = 0.050
*wR*(*F* ^2^) = 0.116
*S* = 1.05
2400 reflections
174 parameters
H-atom parameters constrained
Δρ_max_ = 0.17 e Å^−3^
Δρ_min_ = −0.24 e Å^−3^

### 

Data collection: *SMART* (Bruker, 2000 ▶); cell refinement: *SAINT* (Bruker, 2000 ▶); data reduction: *SAINT*; program(s) used to solve structure: *SHELXS97* (Sheldrick, 2008 ▶); program(s) used to refine structure: *SHELXL97* (Sheldrick, 2008 ▶); molecular graphics: *SHELXTL* (Sheldrick, 2008 ▶); software used to prepare material for publication: *SHELXTL*.

## Supplementary Material

Crystal structure: contains datablocks global, I. DOI: 10.1107/S1600536810050919/im2246sup1.cif

Structure factors: contains datablocks I. DOI: 10.1107/S1600536810050919/im2246Isup2.hkl

Additional supplementary materials:  crystallographic information; 3D view; checkCIF report

## Figures and Tables

**Table 1:** Hydrogen-bond geometry (Å, °)

*D*—H⋯*A*	*D*—H	H⋯*A*	*D*⋯*A*	*D*—H⋯*A*
C2—H2⋯O4^i^	0.93	2.59	3.257 (3)	129
C3—H3⋯O2^ii^	0.93	2.55	3.272 (3)	135

Symmetry codes: (i) 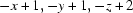; (ii) 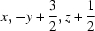.

**Fig. 1.**
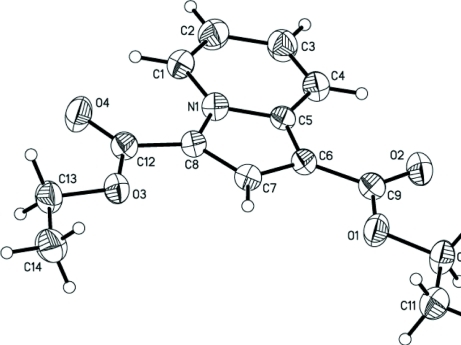
A view of the title compound showing the atom-numbering scheme and displacement ellipsoids drawn at 30% probability level.

**Fig. 2.**
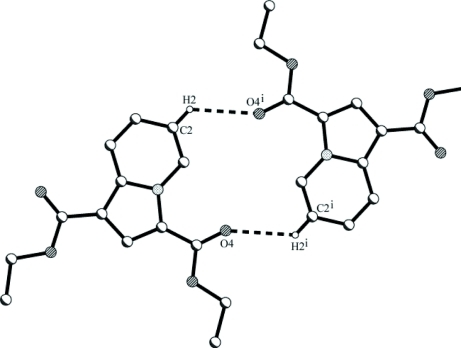
A view of the dimer. Dashed lines indicate weak C—H···O interactions and all H atoms except H2 have been omitted for clarity (i: 1 - x,1 - y,2 - z).

**Fig. 3.**
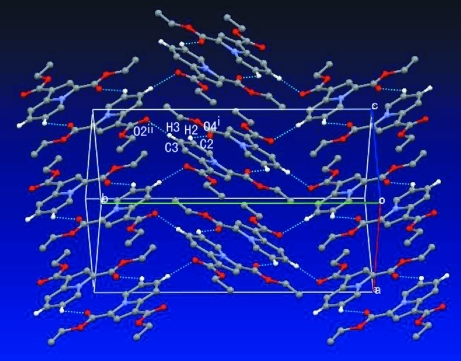
A view of the 3-D packing. Dashed lines indicate weqk C—H···O interaction and all H atoms except H2 and H3 have been omitted for clarity (i: 1 - x,1 - y,2 - z; ii: x,1.5 - y,1/2 + z).

## supplementary crystallographic information

### Comment

Indolizine and their derivatives have been comprehensively applied in biology and medicine due to their particular structures and pharmaceutical properties (Tukulula *et al.*, 2010; James *et al.*, 2008; Teklu *et al.*, 2005). They can also be used as organic fluorescence probes (Shen *et al.*, 2008; Shen *et al.*, 2006). In our continuing studies on organic fluorescence probes, we synthesized diethyl indolizine-1,3-dicarboxylate, the title compound, (I).

The crystal structure of the title compound, C_14_H_15_NO_4_, reveals that all the bond lengths and angles have normal values. As shown in Fig. 1, the molecule is essentially planar. All atoms of the molecule locate on the same least-squares plane (6.9517(0.0017)*X* + 8.0272(0.0048)Y - 3.7352(0.0022)*Z* = 3.8065 (0.0031)), and the r.m.s. deviation of fitted atoms is 0.0479 (3) Å. The crystal packing is established by weak C—H···O interactions. Two neighbouring molecules form a dimer via the weak hydrogen bond C2—H2···O4^i ^(i: 1 - *x*,1 - *y*,2 - *z*) (Fig. 2) with a distance between C2 and O4 of 3.257 (3) Å. Furthermore, the dimers are interconnected to form a 3-D structure by the weak interaction C3—H3···O2^ii^ (ii: *x*,1.5 - *y*,1/2 + *z*) (Fig. 3) with a distance of 3.272 (3)Å between C3 and O2.

### Experimental

Diethyl indolizine-1,3-dicarboxylate was prepared in 24% yield by a 1,3-dipolar cycloaddition from N-(ethoxycarbonylmethy)pyridinium bromide and ethyl acrylate in the presence of NEt_3_ and CrO_3_ in DMF according to a procedure described in the literature (Wang, *et al.*, 2000). Colorless crystals were obtained by recrystallization of the crude product from ethyl acetate at room temperature.

^1^H-NMR (CDCl_3_, 400 MHz) δ: 1.41 (2xt, 6H, 2x-COOCH_2_CH_3_), 4.38 (2xq, 4H, 2x-COOCH_2_CH_3_), 6.97 (ddd, 1H, H_6_), 7.31 (ddd, 1H, H_7_), 8.00 (s, 1H, H_2_), 8.34 (dd, 1H, H_8_), 9.53 (dd, 1H, H_5_).

### Refinement

H atoms were positioned geometrically and refined using a riding model (including free rotation about the ethyl C—C bond), with C—H = 0.93–0.97 Å and with *U*_iso_(H) = 1.2 (1.5 for methyl groups) times *U*_eq_(C).

### Crystal data

**Table:**

C_14_H_15_NO_4_	*F*(000) = 552
*M**_r_* = 261.27	*D*_x_ = 1.314 Mg m^−^^3^
Monoclinic, *P*2_1_/*c*	Mo *K*α radiation, λ = 0.71073 Å
Hall symbol: -P 2ybc	Cell parameters from 1756 reflections
*a* = 7.941 (2) Å	θ = 2.6–22.7°
*b* = 19.700 (4) Å	µ = 0.10 mm^−^^1^
*c* = 8.622 (2) Å	*T* = 291 K
β = 101.770 (3)°	Block, colorless
*V* = 1320.5 (5) Å^3^	0.30 × 0.26 × 0.24 mm
*Z* = 4	

### Data collection

**Table:**

Bruker SMART APEX CCD diffractometer	2400 independent reflections
Radiation source: sealed tube	1567 reflections with *I* > 2σ(*I*)
graphite	*R*_int_ = 0.039
phi and ω scans	θ_max_ = 25.5°, θ_min_ = 2.6°
Absorption correction: multi-scan (*SADABS*; Bruker, 2000)	*h* = −9→9
*T*_min_ = 0.972, *T*_max_ = 0.977	*k* = −23→23
7930 measured reflections	*l* = −10→10

### Refinement

**Table:**

Refinement on *F*^2^	Primary atom site location: structure-invariant direct methods
Least-squares matrix: full	Secondary atom site location: difference Fourier map
*R*[*F*^2^ > 2σ(*F*^2^)] = 0.050	Hydrogen site location: inferred from neighbouring sites
*wR*(*F*^2^) = 0.116	H-atom parameters constrained
*S* = 1.05	*w* = 1/[σ^2^(*F*_o_^2^) + (0.04*P*)^2^ + 0.33*P*] where *P* = (*F*_o_^2^ + 2*F*_c_^2^)/3
2400 reflections	(Δ/σ)_max_ < 0.001
174 parameters	Δρ_max_ = 0.17 e Å^−^^3^
0 restraints	Δρ_min_ = −0.24 e Å^−^^3^

### Special details

**Table:**

Geometry. All e.s.d.'s (except the e.s.d. in the dihedral angle between two l.s. planes) are estimated using the full covariance matrix. The cell e.s.d.'s are taken into account individually in the estimation of e.s.d.'s in distances, angles and torsion angles; correlations between e.s.d.'s in cell parameters are only used when they are defined by crystal symmetry. An approximate (isotropic) treatment of cell e.s.d.'s is used for estimating e.s.d.'s involving l.s. planes.
Refinement. Refinement of *F*^2^ against ALL reflections. The weighted *R*-factor *wR* and goodness of fit *S* are based on *F*^2^, conventional *R*-factors *R* are based on *F*, with *F* set to zero for negative *F*^2^. The threshold expression of *F*^2^ > σ(*F*^2^) is used only for calculating *R*-factors(gt) *etc*. and is not relevant to the choice of reflections for refinement. *R*-factors based on *F*^2^ are statistically about twice as large as those based on *F*, and *R*- factors based on ALL data will be even larger.Least-squares planes (*x*,*y*,*z* in crystal coordinates) and deviations from them (* indicates atom used to define plane)6.9517 (0.0017) *x* + 8.0272 (0.0048) *y* - 3.7352 (0.0022) *z* = 3.8065 (0.0031)* 0.0092 (0.0021) C1 * -0.0460 (0.0022) C2 * -0.0746 (0.0022) C3 * -0.0551 (0.0020) C4 * -0.0046 (0.0019) C5 * 0.0272 (0.0020) C6 * 0.0632 (0.0020) C7 * 0.0674 (0.0020) C8 * 0.0094 (0.0021) C9 * -0.0124 (0.0023) C10 * -0.0150 (0.0021) C11 * 0.0555 (0.0020) C12 * -0.0638 (0.0022) C13 * -0.0916 (0.0022) C14 * 0.0286 (0.0017) N1 * -0.0164 (0.0017) O1 * 0.0319 (0.0017) O2 * 0.0148 (0.0016) O3 * 0.0723 (0.0017) O4Rms deviation of fitted atoms = 0.0479

### Fractional atomic coordinates and isotropic or equivalent isotropic displacement parameters (Å^2^)

**Table:**

	*x*	*y*	*z*	*U*_iso_*/*U*_eq_	
C1	0.3394 (3)	0.57688 (11)	0.8498 (3)	0.0612 (6)	
H1	0.4111	0.5441	0.9062	0.073*	
C2	0.3039 (3)	0.63422 (12)	0.9219 (3)	0.0693 (7)	
H2	0.3508	0.6409	1.0288	0.083*	
C3	0.1963 (3)	0.68414 (12)	0.8365 (3)	0.0690 (7)	
H3	0.1732	0.7237	0.8871	0.083*	
C4	0.1266 (3)	0.67502 (11)	0.6820 (3)	0.0599 (6)	
H4	0.0555	0.7082	0.6263	0.072*	
C5	0.1613 (3)	0.61567 (9)	0.6055 (3)	0.0468 (5)	
C6	0.1093 (3)	0.59243 (10)	0.4502 (3)	0.0524 (6)	
C7	0.1841 (3)	0.52881 (10)	0.4431 (3)	0.0552 (6)	
H7	0.1701	0.5015	0.3534	0.066*	
C8	0.2815 (3)	0.51264 (10)	0.5885 (3)	0.0526 (6)	
C9	−0.0036 (3)	0.62911 (11)	0.3236 (3)	0.0579 (6)	
C10	−0.1463 (3)	0.62404 (12)	0.0531 (3)	0.0710 (7)	
H10A	−0.0954	0.6662	0.0268	0.085*	
H10B	−0.2587	0.6339	0.0752	0.085*	
C11	−0.1616 (4)	0.57481 (13)	−0.0806 (3)	0.0770 (8)	
H11A	−0.0491	0.5640	−0.0982	0.116*	
H11B	−0.2291	0.5946	−0.1748	0.116*	
H11C	−0.2166	0.5341	−0.0549	0.116*	
C12	0.3783 (3)	0.45130 (11)	0.6400 (3)	0.0573 (6)	
C13	0.4462 (3)	0.34094 (11)	0.5611 (3)	0.0698 (7)	
H13A	0.4010	0.3188	0.6445	0.084*	
H13B	0.5689	0.3476	0.5979	0.084*	
C14	0.4129 (4)	0.29840 (12)	0.4151 (4)	0.0864 (9)	
H14A	0.2911	0.2926	0.3791	0.130*	
H14B	0.4662	0.2548	0.4382	0.130*	
H14C	0.4598	0.3204	0.3340	0.130*	
N1	0.2678 (2)	0.56704 (9)	0.6903 (2)	0.0575 (5)	
O1	−0.0371 (2)	0.59243 (8)	0.1895 (2)	0.0692 (5)	
O2	−0.0599 (2)	0.68564 (8)	0.3343 (2)	0.0728 (5)	
O3	0.3616 (2)	0.40572 (7)	0.52177 (19)	0.0630 (5)	
O4	0.4634 (2)	0.44071 (8)	0.7710 (2)	0.0758 (5)	

### Atomic displacement parameters (Å^2^)

**Table:**

	*U*^11^	*U*^22^	*U*^33^	*U*^12^	*U*^13^	*U*^23^
C1	0.0625 (16)	0.0608 (14)	0.0556 (16)	−0.0007 (12)	0.0013 (13)	0.0057 (12)
C2	0.0755 (18)	0.0710 (15)	0.0570 (16)	−0.0005 (13)	0.0032 (14)	−0.0028 (13)
C3	0.0789 (19)	0.0591 (14)	0.0676 (19)	0.0047 (13)	0.0114 (15)	−0.0030 (13)
C4	0.0610 (15)	0.0529 (12)	0.0648 (17)	0.0031 (11)	0.0105 (13)	0.0059 (11)
C5	0.0422 (12)	0.0446 (11)	0.0532 (14)	−0.0059 (9)	0.0090 (11)	0.0060 (10)
C6	0.0486 (14)	0.0472 (11)	0.0598 (16)	−0.0025 (10)	0.0070 (12)	0.0078 (10)
C7	0.0547 (14)	0.0504 (12)	0.0578 (16)	−0.0045 (10)	0.0052 (12)	0.0025 (11)
C8	0.0512 (14)	0.0471 (11)	0.0569 (15)	−0.0010 (10)	0.0050 (12)	0.0060 (10)
C9	0.0558 (15)	0.0535 (13)	0.0628 (17)	−0.0068 (11)	0.0080 (13)	0.0096 (12)
C10	0.0721 (18)	0.0696 (15)	0.0657 (18)	0.0119 (13)	0.0011 (14)	0.0191 (13)
C11	0.0823 (19)	0.0765 (16)	0.0643 (18)	−0.0001 (14)	−0.0039 (14)	0.0078 (14)
C12	0.0528 (15)	0.0543 (13)	0.0637 (17)	−0.0017 (11)	0.0097 (13)	0.0081 (12)
C13	0.0644 (16)	0.0550 (13)	0.089 (2)	0.0107 (12)	0.0126 (15)	0.0112 (13)
C14	0.084 (2)	0.0547 (14)	0.115 (3)	0.0083 (13)	0.0095 (18)	−0.0047 (15)
N1	0.0557 (12)	0.0539 (10)	0.0606 (13)	−0.0007 (9)	0.0066 (10)	0.0058 (10)
O1	0.0786 (12)	0.0619 (9)	0.0586 (11)	0.0126 (8)	−0.0062 (9)	0.0075 (8)
O2	0.0819 (12)	0.0518 (9)	0.0797 (13)	0.0090 (8)	0.0048 (10)	0.0100 (8)
O3	0.0681 (11)	0.0469 (8)	0.0696 (11)	0.0067 (7)	0.0036 (9)	0.0061 (8)
O4	0.0801 (13)	0.0756 (11)	0.0644 (12)	0.0177 (9)	−0.0023 (10)	0.0116 (9)

### Geometric parameters (Å, °)

**Table:**

C1—C2	1.346 (3)	C9—O1	1.344 (3)
C1—N1	1.390 (3)	C10—O1	1.451 (3)
C1—H1	0.9300	C10—C11	1.492 (3)
C2—C3	1.408 (3)	C10—H10A	0.9700
C2—H2	0.9300	C10—H10B	0.9700
C3—C4	1.346 (3)	C11—H11A	0.9600
C3—H3	0.9300	C11—H11B	0.9600
C4—C5	1.397 (3)	C11—H11C	0.9600
C4—H4	0.9300	C12—O4	1.210 (3)
C5—N1	1.384 (3)	C12—O3	1.344 (3)
C5—C6	1.395 (3)	C13—O3	1.450 (2)
C6—C7	1.394 (3)	C13—C14	1.490 (3)
C6—C9	1.455 (3)	C13—H13A	0.9700
C7—C8	1.369 (3)	C13—H13B	0.9700
C7—H7	0.9300	C14—H14A	0.9600
C8—N1	1.404 (3)	C14—H14B	0.9600
C8—C12	1.452 (3)	C14—H14C	0.9600
C9—O2	1.211 (3)		
			
C2—C1—N1	119.5 (2)	O1—C10—H10B	110.4
C2—C1—H1	120.3	C11—C10—H10B	110.4
N1—C1—H1	120.3	H10A—C10—H10B	108.6
C1—C2—C3	120.4 (2)	C10—C11—H11A	109.5
C1—C2—H2	119.8	C10—C11—H11B	109.5
C3—C2—H2	119.8	H11A—C11—H11B	109.5
C4—C3—C2	120.5 (2)	C10—C11—H11C	109.5
C4—C3—H3	119.8	H11A—C11—H11C	109.5
C2—C3—H3	119.8	H11B—C11—H11C	109.5
C3—C4—C5	119.9 (2)	O4—C12—O3	122.9 (2)
C3—C4—H4	120.1	O4—C12—C8	126.1 (2)
C5—C4—H4	120.1	O3—C12—C8	111.1 (2)
N1—C5—C6	108.02 (18)	O3—C13—C14	107.7 (2)
N1—C5—C4	119.2 (2)	O3—C13—H13A	110.2
C6—C5—C4	132.8 (2)	C14—C13—H13A	110.2
C7—C6—C5	107.0 (2)	O3—C13—H13B	110.2
C7—C6—C9	128.1 (2)	C14—C13—H13B	110.2
C5—C6—C9	125.0 (2)	H13A—C13—H13B	108.5
C8—C7—C6	109.6 (2)	C13—C14—H14A	109.5
C8—C7—H7	125.2	C13—C14—H14B	109.5
C6—C7—H7	125.2	H14A—C14—H14B	109.5
C7—C8—N1	107.08 (18)	C13—C14—H14C	109.5
C7—C8—C12	129.7 (2)	H14A—C14—H14C	109.5
N1—C8—C12	123.2 (2)	H14B—C14—H14C	109.5
O2—C9—O1	123.3 (2)	C5—N1—C1	120.57 (19)
O2—C9—C6	125.5 (2)	C5—N1—C8	108.35 (18)
O1—C9—C6	111.2 (2)	C1—N1—C8	131.08 (19)
O1—C10—C11	106.8 (2)	C9—O1—C10	116.56 (18)
O1—C10—H10A	110.4	C12—O3—C13	116.16 (19)
C11—C10—H10A	110.4		
			
N1—C1—C2—C3	−0.4 (4)	C7—C8—C12—O3	−1.1 (3)
C1—C2—C3—C4	0.4 (4)	N1—C8—C12—O3	176.12 (18)
C2—C3—C4—C5	−0.1 (4)	C6—C5—N1—C1	179.92 (18)
C3—C4—C5—N1	−0.2 (3)	C4—C5—N1—C1	0.1 (3)
C3—C4—C5—C6	−179.9 (2)	C6—C5—N1—C8	−0.6 (2)
N1—C5—C6—C7	0.7 (2)	C4—C5—N1—C8	179.61 (18)
C4—C5—C6—C7	−179.5 (2)	C2—C1—N1—C5	0.2 (3)
N1—C5—C6—C9	−179.89 (19)	C2—C1—N1—C8	−179.2 (2)
C4—C5—C6—C9	−0.2 (4)	C7—C8—N1—C5	0.3 (2)
C5—C6—C7—C8	−0.6 (2)	C12—C8—N1—C5	−177.48 (19)
C9—C6—C7—C8	−179.9 (2)	C7—C8—N1—C1	179.6 (2)
C6—C7—C8—N1	0.2 (2)	C12—C8—N1—C1	1.9 (3)
C6—C7—C8—C12	177.7 (2)	O2—C9—O1—C10	0.2 (3)
C7—C6—C9—O2	−176.9 (2)	C6—C9—O1—C10	−178.95 (19)
C5—C6—C9—O2	3.9 (4)	C11—C10—O1—C9	178.84 (19)
C7—C6—C9—O1	2.2 (3)	O4—C12—O3—C13	2.4 (3)
C5—C6—C9—O1	−177.01 (19)	C8—C12—O3—C13	−177.22 (18)
C7—C8—C12—O4	179.3 (2)	C14—C13—O3—C12	−179.72 (19)
N1—C8—C12—O4	−3.5 (4)		

### Hydrogen-bond geometry (Å, °)

**Table:**

*D*—H···*A*	*D*—H	H···*A*	*D*···*A*	*D*—H···*A*
C2—H2···O4^i^	0.93	2.59	3.257 (3)	129
C3—H3···O2^ii^	0.93	2.55	3.272 (3)	135

Symmetry codes: (i) −*x*+1, −*y*+1, −*z*+2; (ii) *x*, −*y*+3/2, *z*+1/2.
